# Hair Specialist, Trichologist or Dermato-Trichologist?

**DOI:** 10.4103/0974-7753.77530

**Published:** 2010

**Authors:** Patrick Yesudian

**Affiliations:** President, The hair research society of India, Chennai, India

When I was a school boy my mother used to send me to the end of the road where Kuppan, the barber, used to cut my hair in a nook 8 ft by 6 ft in size, with no name boards outside. The shop had just two chairs with mirrors in front and Kuppan had one assistant to help him. Then I went to college and I still used to visit Kuppan’s shop for my monthly hair cuts. However, now there were three chairs, two assistants, and many gadgets to help him trim the hair. The prominent board outside the entrance gleamed ‘Hair Dressing saloon’ and Kuppan no longer called himself a barber; he was a hair dresser, a term used by the Webster’s dictionary to define a Trichologist.

Years rolled by and after a stint abroad I went back for my hair cut, although the need for it had drastically come down due to the onset of Hamilton Grade V androgenetic alopecia (which is now beyond VII — what do you call it?) As I stood outside the shop I could hardly recognize the beautiful marble façade and neon board claiming it to be a ‘Beauty Salon’. The shop had now become a ‘Spa,’ with extended chairs and uniformed male and female assistants. Hair cutting had taken a back seat with hair coloring, hair straightening, hair waving, hair weaving, and many other money spinning hair gimmicks that were being provided. An expert was giving special advice on how to treat alopecia areata, seborrhoeic dermatitis, and hair fall, with attractive package offers and to my great surprise, it was Kuppan himself, only now his name was K.U. Pan! The barber of yesteryear had become the Trichologist of today. I am not decrying the term ‘barber’ because in the history of medicine, surgeons evolved from barbers!

What I am trying to convey from the above anecdote is that the term ‘trichology’ has become associated with cosmetology. Therefore, as experts delving into scientific research and an in-depth study of the different aspects of this skin appendage, we should call ourselves DERMATO TRICHOLOGISTS. This definition would put an end to the present terminological confusion.

